# Transcriptomic and Proteomic Study on the High-Fat Diet Combined With AOM/DSS-Induced Adenomatous Polyps in Mice

**DOI:** 10.3389/fonc.2021.736225

**Published:** 2021-08-26

**Authors:** Cui Guo, Yimin Xu, Xinyue Han, Xiaoqiang Liu, Runnan Xie, Zhihong Cheng, Xiaoling Fu

**Affiliations:** ^1^Second Department of Oncology, Yueyang Hospital of Integrated Traditional Chinese and Western Medicine, Shanghai University of Traditional Chinese Medicine, Shanghai, China; ^2^Liaoning University of Traditional Chinese Medicine, Shenyang, China; ^3^Department of Pain, Shibei Hospital, Shanghai, China; ^4^Department of Traditional Chinese Medicine, Miaohang Town Community Health Service Center, Shanghai, China; ^5^China State Institute of Pharmaceutical Industry, National Pharmaceutical Engineering Research Center, Shanghai, China

**Keywords:** colorectal adenoma, AOM/DSS, transcriptomics, proteomics, colorectal cancer

## Abstract

**Objective:**

To screen and identify molecular targets and bacteria genus leading to adenomatous polyps in mouse induced by high-fat diet (HFD) +AOM/DSS using omics technology.

**Methods:**

The molecular targets of colorectal adenoma disease were obtained from the GeneCards and OMIM database. The SPF C57BL mice were randomly divided into blank (Control) and AOM/DSS+HFD colorectal adenoma model (ADH) groups. The ADH model group was intraperitoneally injected with AOM reagent. Then, mice were given with 2.5% DSS (in free drinking water) and high-fat diet to establish the mouse model. During this period, the changes of physical signs of mice in each group were observed. After the end of modeling, HE staining was used to evaluate the histopathological change of mice. The differentially expressed genes and proteins in the Control group and ADH group were detected by RNA-seq transcriptome sequencing and Tandem Mass Tags (TMT) quantitative proteomics. The histological results were analyzed by intersection with the intestinal adenoma molecular targets obtained from the database. Moreover, the changes of intestinal flora in the two groups were examined. The correlation between targets and differential bacteria was analyzed and verified by Parallel Reaction Monitoring (PRM) to comprehensively evaluate the mouse model of adenomatous polyp induced by AOM/DSS+HFD.

**Results:**

The general condition and histopathological results of mice confirmed that the ADH mouse model was successfully established and tubular adenoma was formed. A total of 604 genes and 42 proteins related to intestinal adenoma were obtained by histological analysis and database intersection analysis. The intestinal microflora of ADH mice was different from that of normal mice, and the constituents and abundance of intestinal flora were similar to those of human intestinal adenoma. GATA4 and LHPP were selected as potential pathological markers of the model mice by correlation analysis of targets and intestinal flora. The results of PRM verification were highly consistent with the results of RNA-Seq transcriptome sequencing and TMT analysis.

**Conclusion:**

The pathological results, molecular pathological markers and the changes of intestinal flora suggest that the mouse ADH model is ideal for studying the transformation of inflammatory cancer. The ADH model will be helpful for understanding the occurrence and development of human colorectal cancer at the transcriptomic and proteomic level.

## Introduction

Colorectal cancer (CRC) is one of the most common gastrointestinal malignancies with the third highest morbidity and mortality worldwide (1). Colorectal adenoma (CRA) is a protruding lesion originating from the epithelium of the colorectal mucosa and a precancerous lesion of CRC with high recurrence and carcinogenesis (2). More than 90% of CRC are caused by CRA carcinogenesis (3). According to the pathological classification, CRA can be divided into tubular adenoma, villous adenoma, villous tubular adenoma and serrated adenoma (4), among them, the incidence of tubular adenoma accounts for 75-80% of all adenomas (5). A number of studies have confirmed that genetics, inflammatory disease, lipid metabolic disorders, and sugar metabolic disorders are risk factors for the onset of colorectal adenomas (6). At present, the adenoma model of inflammatory cancer transformation is one of the commonly used models to study the carcinogenesis of CRC (7, 8). Research on the pathogenesis and therapeutic mechanism of the disease is limited by time, space and ethics (9). The establishment of human disease simulated animal model has important scientific value, and the use of animal model for experimental research is very important (10), which is convenient and effective in understanding the mechanisms of disease occurrence and development and research on prevention and control measures, and can avoid the risk brought by human experiments (11).

In the animal models of colorectal precancerous lesions, it is known that the mouse mode of colitis is related to inflammatory cancer transformation (12). AOM, the DNA alkylation product of chemical carcinogen 1,2-dimethylhydrazine, can be injected intraperitoneally and metabolized through bile. AOM can be further activated by intestinal flora metabolism to cause cancer. DSS is an inflammatory chemical agent. Animal drinking water containing DSS can create an inflammatory bowel disease model (13). The mouse colon cancer model established by the combination of AOM and DSS can simulate the whole process of normal mucosa – inflammation – tumor formation. At present, the AOM/DSS compound chemical method is commonly used to establish the colorectal adenoma animal model (14, 15). The mouse model simulates the physiological and pathological process of cancer induced by human chronic intestinal inflammation, and is an effective tool to study the mechanism of development of colorectal tumor in inflammatory environment (16). In recent years, with the improvement of living standards, high-fat diet is becoming more and more common (17). Relevant epidemiological studies have shown that high-fat diet is closely related to colorectal cancer (18–20). Colorectal adenoma is usually detected by proctoscopy. If simple biochemical means can be used in the general survey, it can save a lot of manpower and time. Undoubtedly, the biochemical markers of adenoma are of great significance to clarify the mechanism of adenoma evolution into cancer. However, the occurrence of intestinal adenoma is not limited to a single gene mutation, but also involves DNA modification, oncogenes and tumor suppressor genes. Currently, there was no study on the transcriptomic and proteomic levels on CRA animal models, and the evaluation criteria on RNA and protein are incomplete (21).

In the current study, the inflammation-related intestinal adenoma model induced by AOM/DSS combined with high-fat diet was established. We comprehensively analyze the ADH model from the aspects of histopathology, genes, proteins and intestinal flora, to explore the biomarkers related to the pathogenesis of the disease. This study provides a new idea for studying animal modeling of CRA at the transcriptomic and proteomic level.

## Materials and Methods

### Animal

Six SPF grade C57BL/6 mice, male, aged 7 to 8 weeks and weighing 17 to 19 g, were purchased from Shanghai Jihui Experimental Animal feeding Co., Ltd., certificate number: 20170012005900. Mice were adapted in an independent ventilation cage for 1 week in a constant temperature 23 ± 2°C, constant humidity 50% ± 10% and 12 hours of day and night cycle. This experiment was approved by the Experimental Animal Ethics Committee of Shanghai University of Traditional Chinese Medicine (Ethics No. YYLAC-2019-042-1).

### Animal Modeling

**Figure 1:**
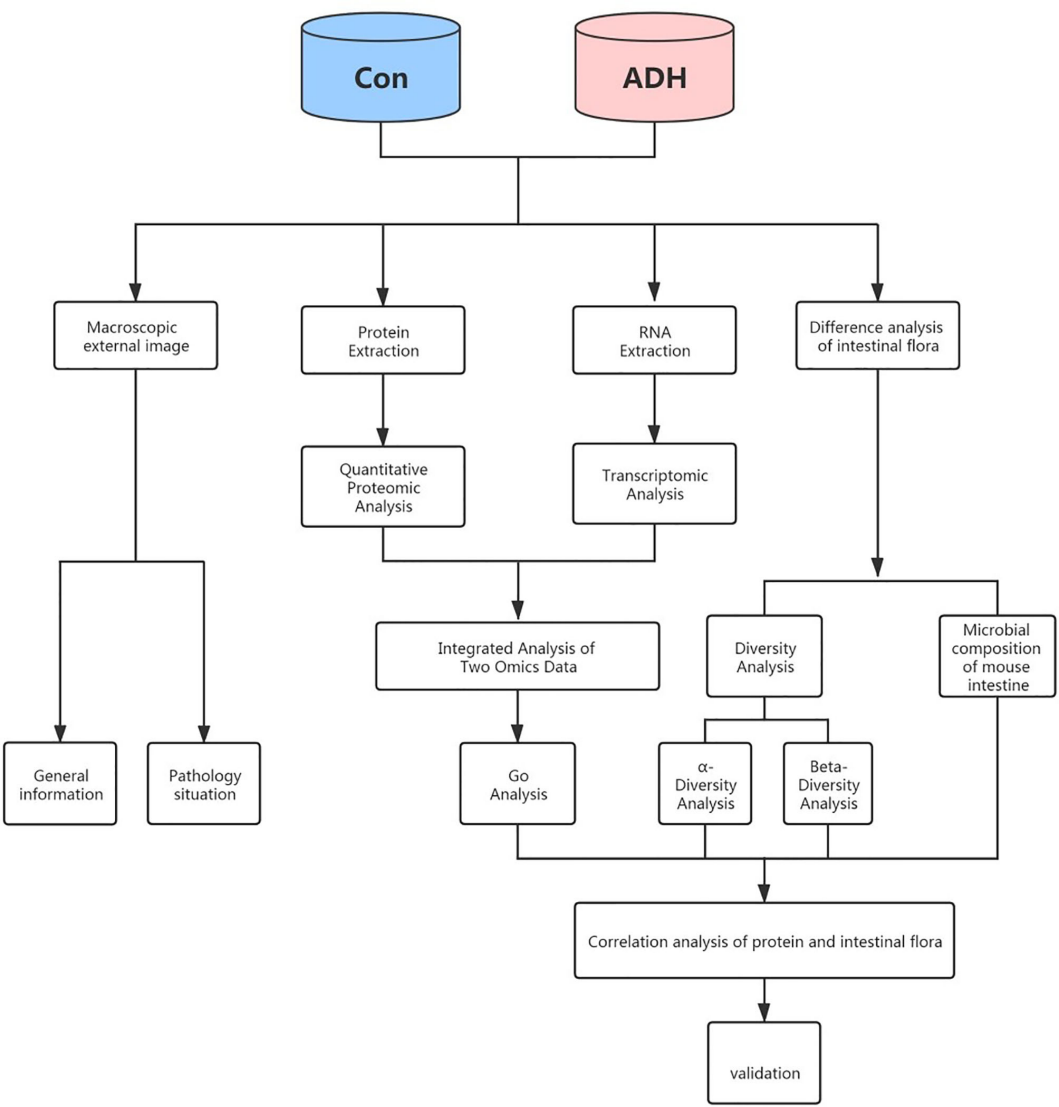
The overall workflow of this study.

**Figure 2:**
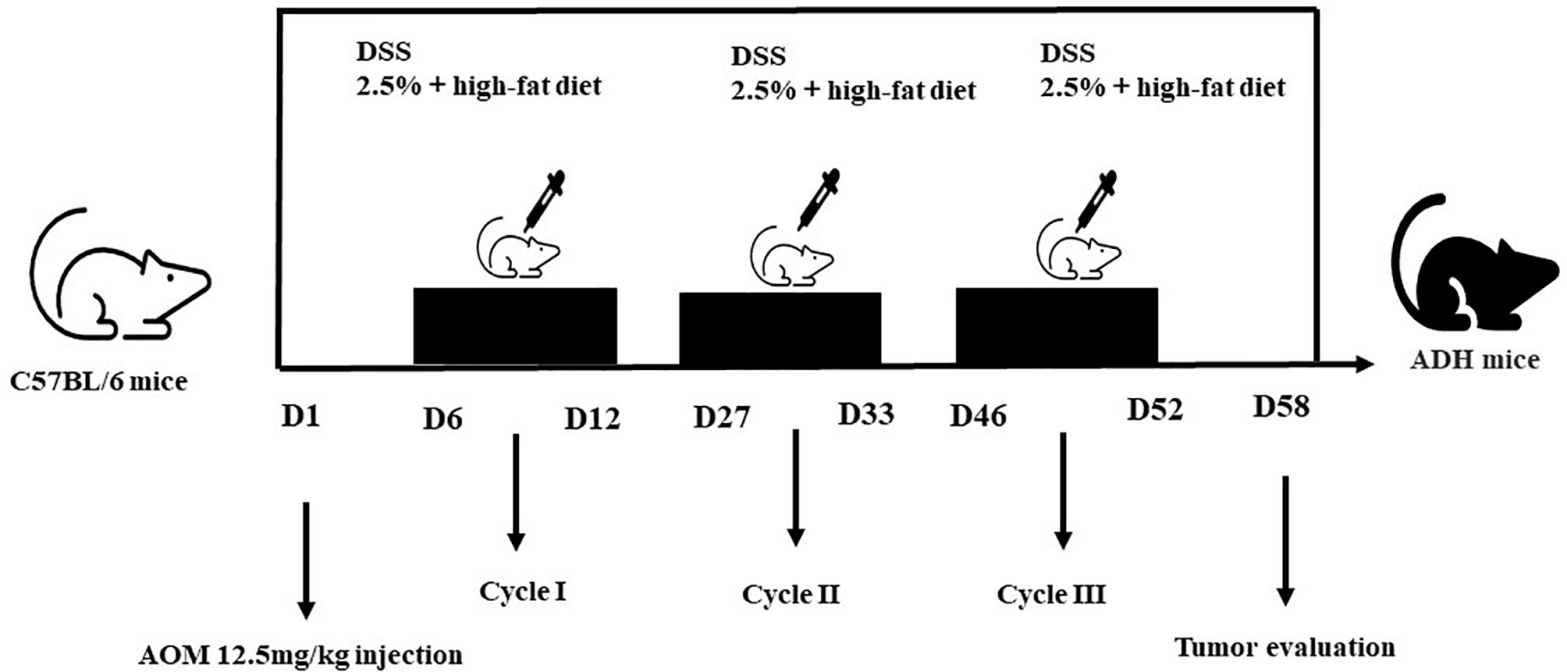
Diagram shows the experimental course of HFD+AOM/DSS mouse model.

The study process is outlined in **Figure 1**. The C57BL/3 mice of 8-9 weeks were intraperitoneally injected with AOM (12.5mg/kg) (Ameresco)reagent on the first day of the experiment. On the 6th, 27th and 46th day of the experiment, mice were administered 2.5% DSS (sigma) drinking water for 5 days. Mice were given routine aqueous solution provided by the laboratory at other times. At the same time, they were fed with a high-fat diet to establish the AOM/DSS+HFD -induced intestinal adenoma (ADH) animal model (**Figure 2**). The normal diet consisted of a standard laboratory chow (NIH-41 open formula diet; Zeigler Bros., Inc., Gardners, PA, USA) with 5% fat, whereas the HFD contained 45% fat (D12451 open formula diet; Research Diets, Inc., New Brunswick, NJ, USA). During the whole experiment, feed and water were given regularly, and the body weight was weighed once a week. At the time when the mice were given DSS aqueous solution, the nutritional status, hair, appetite, activity status, stool morphology and the presence of occulted blood or visible bloody stool were observed every day. During this period, the changes in the physical signs of mice in each group were observed, and the body weight of mice was recorded. After the modeling, the colon tissue was dissected. The changes of the colon of mice in each group, the occurrence of adenomas in the small intestine and colon of mice were observed, and HE histopathological staining was performed.

### RNA Sequencing and Differential Expression Gene Analysis

The eukaryotic mRNA sequencing is based on the Illumina Novaseq 6000 sequencing platform. All the mRNA transcribes were sequenced using the Illumina TruseqTM RNA sample prep Kit. The FASTQC software is applied to quality control of the sequence of RNA sequencing, and the known IlluminaTruSeq joint sequence, low quality sequence and ribosome RNA sequence was removed. The reserved sequence is retained to the mouse reference genome using Hisat2, and the StringTie is screened with the reserved sequence, the gene count is normalized by TMM, and the FPKM calculation is performed with the Perl script. The differential gene expression between the model group and the control group was analyzed by EDGER. The P value of <0.05 was considered as of significant difference.

### Analysis of Differentially Expressed Proteins

Protein s were extracted from intestinal tissue, digested with trypsin, and labelled with TMT reagents. The pooled peptides were separated into 15 fractions using a C18 column (Waters BEH C18 4.6×250 mm, 5 µm) on a Rigol L3000 HPLC. When the protein abundance ratio is 1.2 times or more, and P < 0.05, the protein can be considered as a differential protein.

### Screening Colorectal Adenoma-Related Disease Target

OMIM database (https://www.omim.org/) and GeneCards database (https://www.genecards.org/) were used to collect known and verified CRA-related target genes by searching the keyword “Colorectal adenoma”. GO analysis and KEGG analysis were carried out by using the String (http://www.string.db.org/).

### Correlation Analysis

Based on the above results, bioinformatics comparison analysis was carried out. The multiple of gene expression difference >2, P value <0.05, the protein expression multiple >1.5, P value <0.05 were taken as the standards. The differentially expressed genes and proteins were screened for association analysis, and the Person correlation coefficient was calculated.

### Analysis of the Changes of Intestinal Microflora and Its Correlation With Differentially Expressed Proteins

The intestinal microflora of mice was quantitatively and qualitatively detected by the 16SrRNA high-throughput sequencing technology, and the diversity and abundance of intestinal flora between the model and control mice were analyzed. The correlation between differential proteins and phylum-to-genus level differential bacteria was analyzed, and the key targets were screened.

### Validation of Omics Results

The selected targets were analyzed using the GEPIA database (http://gepia.cancer-pku.cn/). Protein quantification was performed using the calibration curve and optical density values of the protein samples. After quantification, 100 µg of the protein samples was digested using trypsin (Promega, United States) in a ratio of protein: trypsin at 50:1. The protein samples were digested at 37°C for 12–16 h. Then, 6 intestinal tissue samples were tested by liquid phase tandem mass spectrometry (LC-MS). Briefly, the C18-reversed phase column (75 μm x 25 cm, Thermo,USA) as equilibrated with solvent A (A:2% formic acid with 0.1% formic acid) and solvent B (B: 80% ACN with 0.1% formic acid). The peptides were eluted using the following gradient: 0-4 min, 0%-5% B; 4-66 min, 5%−23%B; 66-80 min, 23%−29% B; 80−89 min,29%−38% B; 89-91 min, 38-48% B; 91-92 min, 48-100% B; 92-105min, 100% B; 105-106min, 100-0% B) at a flow rate of 300nL/min. The Q Exactive Plus was operated in the data-dependent acquisition mode (DDA) to automatically switch between full scan MS and MS/MS acquisition. The survey of full scan MS spectra (m/z 350-1300) was acquired in the Orbitrap with 70000 resolution. The automatic gain control (AGC) target at 3e6 and the maximum fill time was 20 ms. Then the top 20 most intense precursor ions were selected into collision cell for fragmentation by higher-energy collision dissociation (HCD). The MS/MS resolution was set at 35000 (at m/z 100), the automatic gain control (AGC) target at 1e5, the maximum fill time at 50 ms, and dynamic exclusion was 18 seconds. Peak extraction was performed on the original PRM data using SKYLINE. 3~4 ions with higher abundance from Y3 to YN-1 were selected for quantitative analysis, and manual inspection and correction were carried out. The peak area results of each peptide segment after SKYLINE analysis were derived, including the target peptide sequence, target protein name, and the peak area of each peptide segment used for quantitative analysis. The peak area of the daughter ion of the peptide in the target protein was analyzed.

### Statistical Analysis

SPSS 21 was used for statistical analysis, and the experimental data were expressed in the form of “mean ± standard error” (X_ ± s). Student’s t test was used, and P<0.05 was considered statistically significant.

## Results

### Mouse Adenoma Model

**Figure 3:**
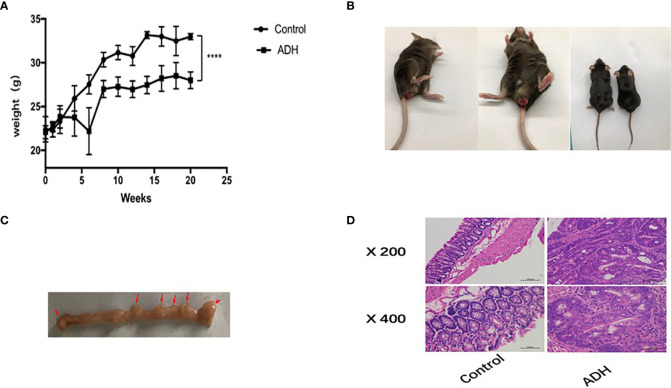
General situation of mice. **(A)** Weight change of mice in the Control group and ADH group; **(B)** General condition of model mice; **(C)**. Intestinal adenoma in ADH group, The red arrow refers to adenoma; **(D)**. Results of HE staining in colonic tissues of each group. ****P < 0.001.

The diet and defecation of the mice in the control group were normal in the entire process of experiments. After three cycles of intraperitoneal injection of AOM and DSS drinking water combined with high-fat diet, the mental state of the model mice was depressed in varying degrees, and the appetite decreased. Some mice died of severe flatulence, and the cause of death was most likely due to acute intestinal obstruction. In the later stage of the experiment, the model mice showed weight loss, thin stool, and even prolapse of anus and hematochezia. No intestinal inflammation and intestinal adenoma formation were observed in the control mice. Large colonic adenoma and adenomas in the small intestine were seen in mice of in the ADH group. The colon was enlarged, and colonic length in the ADH mice was significantly shorter than that in the control group (**Figures 3A–C**). There was no intestinal inflammation and no adenoma formation in the Control group. In the ADH group, there were obvious hyperemia and edema and adenomas of different sizes, especially in the distal colon and rectum. The number of intestinal adenomas in mice was shown in **Table 1**. The size statistics of small intestine and colon adenomas were shown in **Table 2**. There were statistically significant differences in the formation of small intestinal and colorectal adenomas between the Control group and the ADH group (P<0.05).

**Table 1:** The number of adenomas in the range of 1 ~ 3mm and larger than 3mm in the intestinal tract of mice.

Group	Number of adenomas ($\bar{x}$± s)	
	1～3mm	>3mm
Control	0.00	0.00
ADH	5.18±1.60*^#^	1.95±1.02*#

*P < 0.05 VS ADH ; #P > 0.05 VS ADH; *^#^P < 0.05 VS Control.

**Table 2:** Adenoma size of small intestine and colon (mm).

Group	Adenoma size ($\bar{x}$± s)	
	small intestine (mm)	colon (mm)
Control	0.00	0.00
ADH	1.42±0.25*	2.25±1.9*

*P < 0.05 VS Control; P > 0.05 VS ADH.

Histopathologic examination revealed that the colorectal tissue of control group was ruddy, no ulcer bleeding and no granuloma formation. The glandular structure was normal, the mucosal structure was clear, there was no mucosal ulcer, and the glandular epithelial cells had no obvious atypia. In the ADH group, the colon tissue showed dense glands, sieve pore-like structure, obvious atypical hyperplasia of glandular epithelial cells, obvious nuclear enlargement and pathological mitosis, and chronic inflammatory cell infiltration in the stroma, consistent with the signs of adenoma (tubular adenoma)(**Figure 3D**).

### Differential Gene Expression Analysis

**Figure 4:**
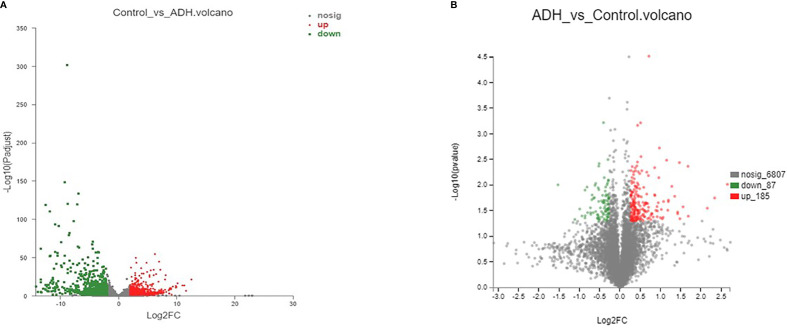
Number of differentially expressed genes and proteins **(A)**. mRNA volcanogram of differential expression; **(B)**. proteins volcanogram of differential expression.

In order to understand the pathogenesis of ADH model, we performed RNA sequencing analysis to obtain the mRNA expression in samples of Control group and ADH group. Using bioinformatics analysis technology, 3423 differential genes were screened out (**S Table 1** and **Figure 4A**). Among them, 1637 genes have low expression, while 1786 genes have high expression in the model group compared with the control group. In the Gene Cards and OMIM databases, 3637 targets related to intestinal adenoma were found. 604 CRA-related genes were screened out by the intersection analysis of differential genes and colorectal adenoma targets obtained from the database (**S Table 2**). Enrichment analysis results showed that differentially expressed genes were significantly enriched in biological processes such as signal transduction, invasive response negative regulation of antigenic process and cell proliferation. These genes were mainly involved in signaling pathways of cancers, PI3K-Akt signaling pathway, MAPK signaling pathway, MicroRNAs in cancer, and others (**S Table 3**)

### Differential Protein Expression Analysis

The TMT-based proteomic method was used to detect differentially expressed proteins in the two groups. A total of 272 differential proteins were screened between the Control group and ADH group (**S Table 4**). The results in **Figure 4B** showed that 87 proteins were expressed at low level, and 185 proteins were highly expressed in the Control group compared with the ADH group. 42 proteins related to CRA were screened by target intersection analysis of differential proteins and colorectal adenoma diseases obtained from the database. The enrichment results showed that the differentially expressed proteins were significantly enriched in biological processes such as signal transduction, positive regulation of transcription and apoptotic process. These proteins mainly involve in signaling pathways such as Regulation of actin cytoskeleton, Leukocyte transendothelial migration and Cell adhesion molecules (CAMs) (**S Table 5**).

### Correlation Analysis of Proteome and Transcriptome

**Figure 5:**
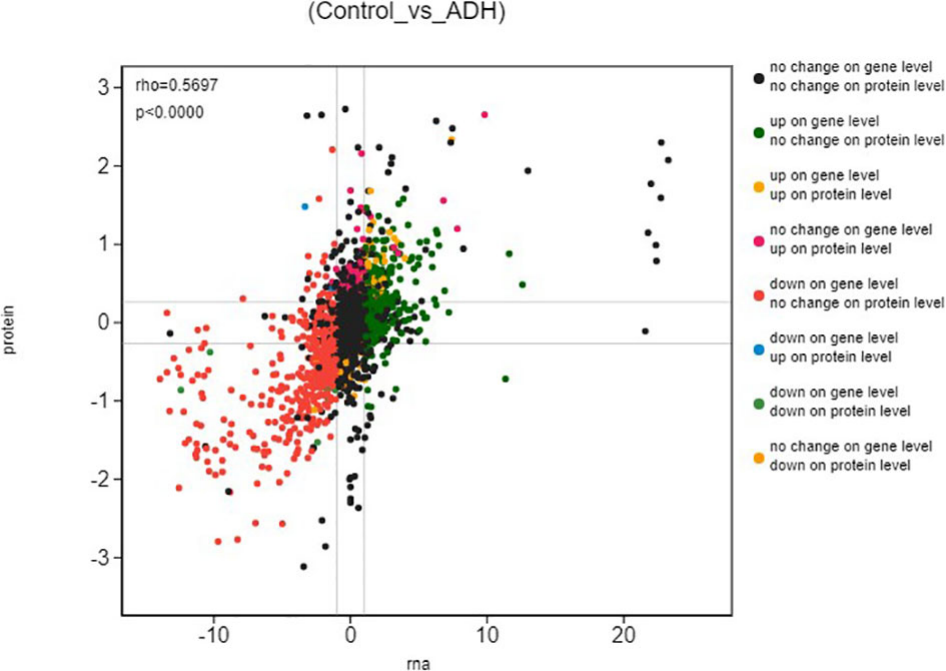
Differential expression analysis (Control_*vs*_ADH) The ordinate represents the expression of multiple proteins in a pair of comparison groups, the log2 (ratio of protein), the abscissa represents the expression of multiple corresponding transcript in the comparison group, the log2 (ratio of gene), logarithm difference takes the logarithmic value respectively; each point represents a protein and its associated transcript, the upper left corner of the picture rho represents the Pearson correlation coefficient between the two groups, p represents the correlation test p value; rho < 0 indicates negative correlation; rho > 0 indicates positive correlation; rho=0 indicates no correlation; the greater the rho, the greater the correlation between the two groups.

Based on the mRNA and protein expression results, the correlation between proteome and transcriptome in the Control and ADH groups was analyzed. The results showed that the correlation coefficient between differential proteins and genes was 0.5697, indicating positively correlated (**Figure 5**). The crossover analysis of histological results showed that 14 genes changed significantly at the transcriptional level, and their encoded proteins also changed (**Table 3**). The trend of change of both mRNAs and proteins was the same.

**Table 3:** Differential targets were obtained by cross-analysis of transcriptome and proteome.

Number	proteome	transcriptome	Target	Up or down regulation
1	ENSMUSP00000034590.2	ENSMUSG00000032085	TAGLN	up
2	ENSMUSP00000025561.7	ENSMUSG00000055114	ANXA1	down
3	ENSMUSP00000031840.7	ENSMUSG00000029816	GPNMB	up
4	ENSMUSP00000061062.7	ENSMUSG00000046733	GPRC5A	up
5	ENSMUSP00000141344.1	ENSMUSG00000022995	ENAH	Up
6	ENSMUSP00000021822.5	ENSMUSG00000021390	OGN	Up
7	ENSMUSP00000021918.8	ENSMUSG00000021464	ROR2	Up
8	ENSMUSP00000079689.5	ENSMUSG00000022150	DAB2	Up
9	ENSMUSP00000067779.4	ENSMUSG00000029094	AFAP1	Up
10	ENSMUSP00000016638.2	ENSMUSG00000016494	CD34	up
11	ENSMUSP00000066927.3	ENSMUSG00000021944	GATA4	down
12	ENSMUSP00000034026.8	ENSMUSG00000031613	HPGD	down
13	ENSMUSP00000113126.1	ENSMUSG00000032081	APOC3	down
14	ENSMUSP00000033241.5	ENSMUSG00000030946	LHPP	down

**Figure 6:**
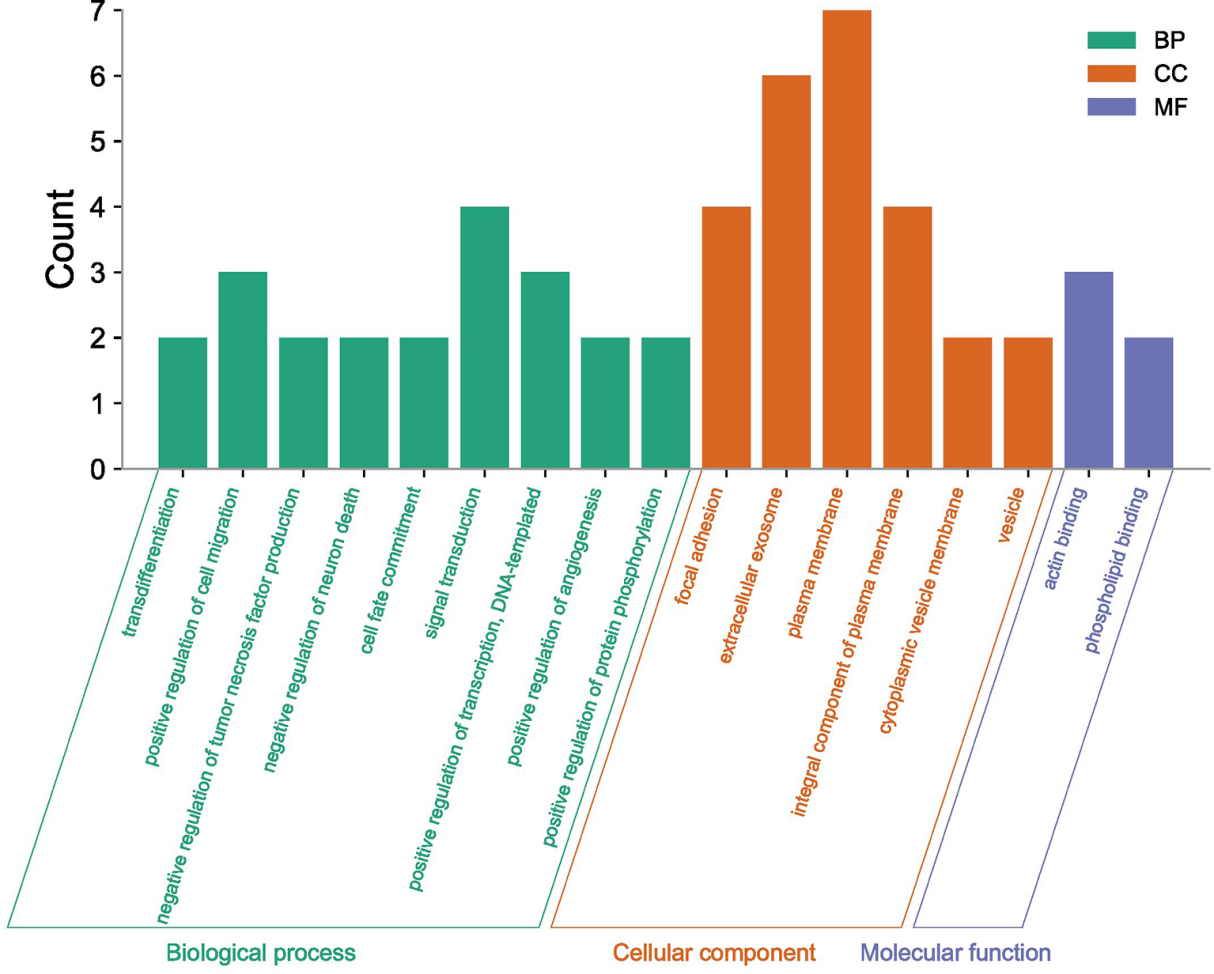
GO enrichment analysis of differential targets.

The fourteen differentially expressed targets were enriched and analyzed in the GO and KEGG pathways, and the enrichment results of three categories (biological processes, molecular functions, cellular components) described by GO first-level classification were obtained. GO enrichment results showed that the differentially expressed targets were significantly enriched in cellular components such as, extracellular exosome, plasma membrane and focal adhesion; in biological processes such as signal transduction, positive regulation of transcription, DNA-templated and positive regulation of cell migration, as well as in molecular functions such as actin binding and phospholipid binding as shown in **Figure 6**, where the longer the bar chart, the more significant the enrichment of differentially expressed targets in this classification or function.

### Diversity Analysis of Intestinal Flora in ADH Model Mice

**Figure 7:**
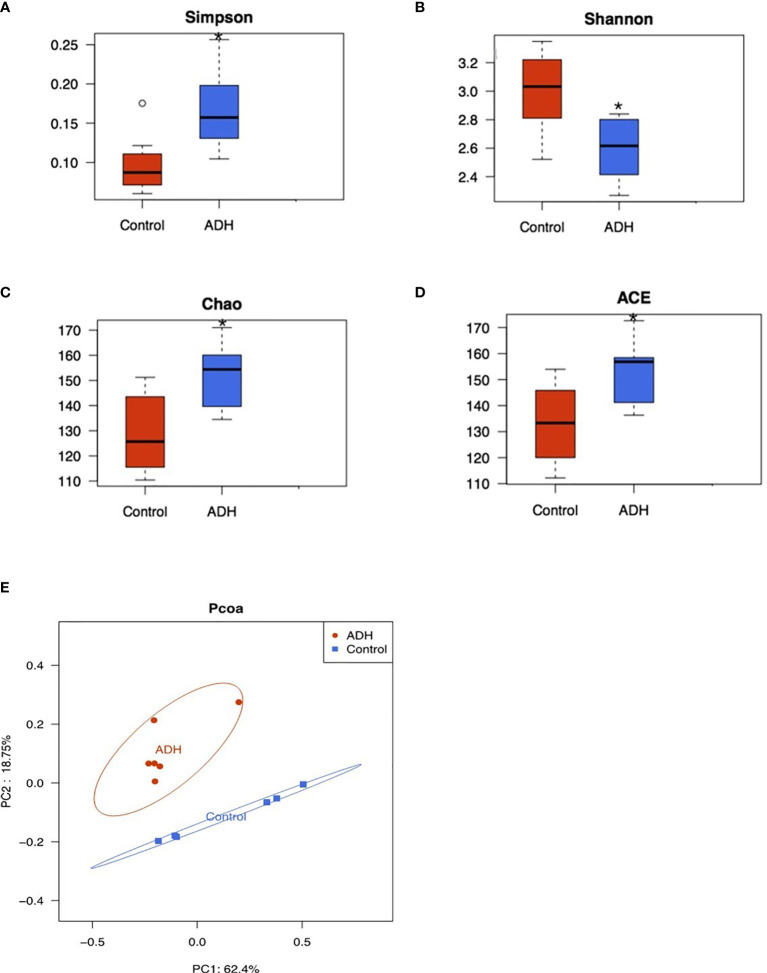
Diversity and composition analysis of intestinal flora. **(A)** Simpson index comparison box chart; **(B)** Box-plot of diversity Shannon index comparison; **(C)** A box chart of Chao index comparison of diversity; DBox-plot of ACE index comparison of diversity; Compared with the Control group,0.05. PCOA analysis of samples; **(D)**. Red represents ADH group and blue represents Control group; **(E)** PCOA analysis of the samples, Red represents ADH group and blue represents Control group. *P < 0.05.

Alpha diversity analysis reflects the changes of intestinal flora abundance and diversity. Chao and Ace can calculate flora abundance, the higher the value, the higher the flora abundance. The Shannon and Simpson can calculate the flora diversity, in which the higher the Shannon value, the higher the flora diversity, while the Simpson index is inversely proportional to the flora diversity. As shown in the **Figures 7A–D**, Simpson index significantly increased, Shannon index significantly decreased, Chao and Ace index also significantly increased in the model group compared with the control group. These changes had statistical significance.

Beta diversity analysis showed that there were significant differences in intestinal flora between ADH group and Control group (**Figure 7D**), distributed along PC1 axis, Control group mainly distributed on the right side, while ADH group mostly distributed on the left side; along PC2 axis, intestinal flora of ADH group distributed above the Control group, indicating that the intestinal flora of Control group and ADH group are different regardless the direction of PC1 or PC2 (**Figure 7E**).

### Analysis of Different Intestinal Floral Species in ADH and Control Mice

At the level of phylum, class, order, family and genus, statistically different bacteria were obtained among each group. In the control group, Actinobacteria, Bifidobacterium (Bifidobacteriales, Bifidobacteriaceae), Olsenella, Atopobiaceae, Verrucomicrobiales (Verrucomicrobia,Verrucomicrobiae), Akkermansia (Akkermansiaceae), Coriobacteriales, Family_XIII_AD3011,Lachnospiraceace_NK4A136 and other bacterial communities played a main role. In the ADH mice, Saccharimonadia (Scharimonadaceae, Scharimonadales), Patescibacteria, Candidatus_Scharimonas,Erysipelotrichaceae, Firmicutes, Ruminococcaceae_UCG-014, Erysipelotrichales, Bacteroidaceae and other bacteria played a major role in the microorganism group.

**Figure 8:**
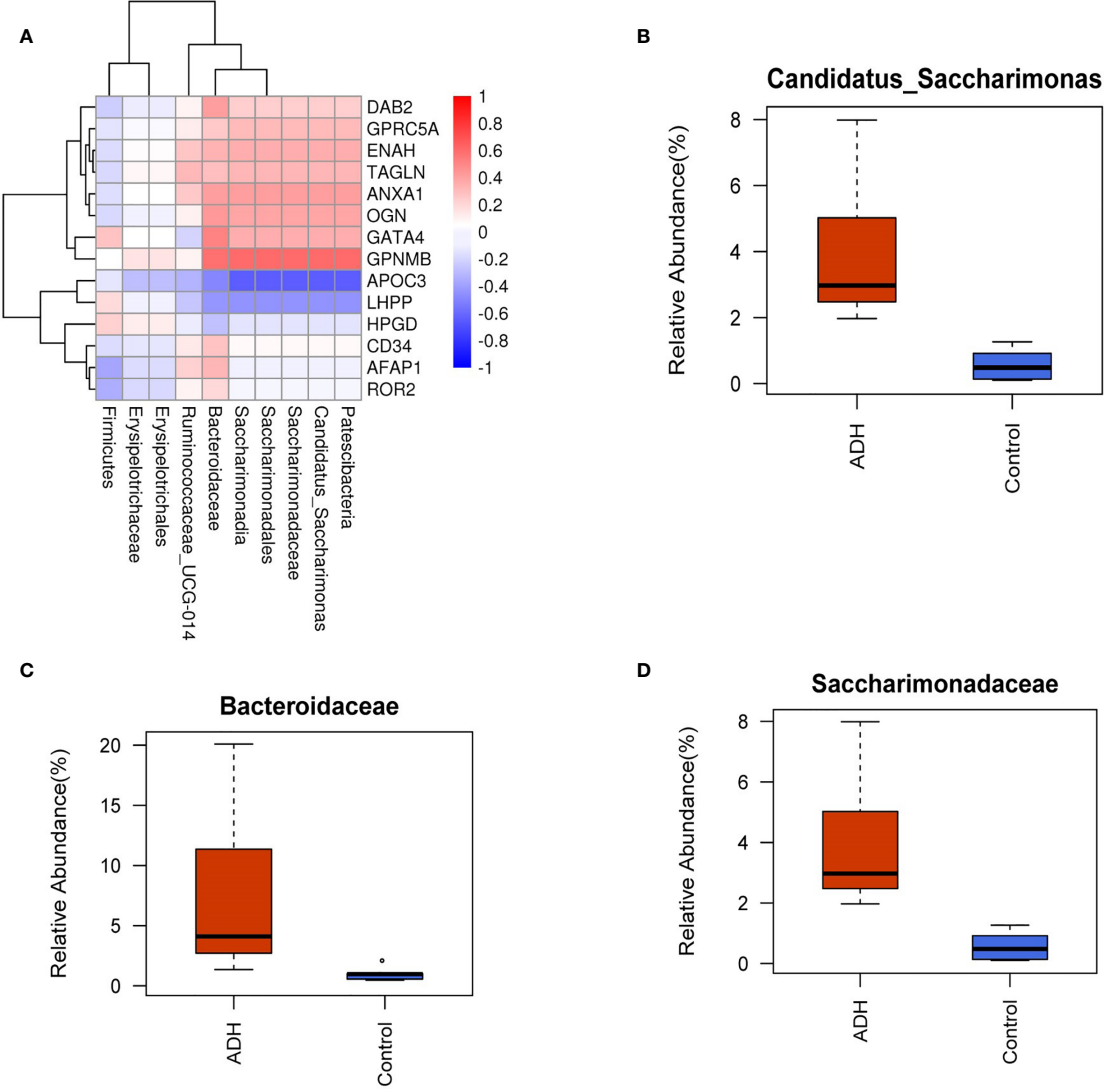
Correlation between protein and intestinal flora. **(A)** Correlation coefficient heat map; **(B–D)** Difference analysis of horizontal intestinal flora of family and genus.

The correlation analysis between 14 differential proteins and differential bacteria in the model group showed that the correlation coefficients of APOC3, GPNMB, GA TA4 and LHPP were high (**Figure 8A**). At the level of family and genus, the abundance of Candidatus_saccharimonas, Saccharimonadales and Bacteroidaceae in ADH mice increased significantly (**Figures 8B–D**). There was a negative correlation between Saccharimonadaceae, Candidatus_Saccharimonas and APOC3, and a positive correlation between Saccharimonadaceae, Candidatus_Saccharimonas and GPNMB, a positive correlation between Bacteroidaceae and GATA4, and a correlation coefficient of 0.58 between Saccharimonadales, Candidatus_Saccharimonas and LHPP, and a negative correlation between Saccharimonadales, Candidatus_Saccharimonas and LHPP with a correlation coefficient of 0.44 (**S Table 6**).

### Data Reliability Analysis

**Figure 9:**
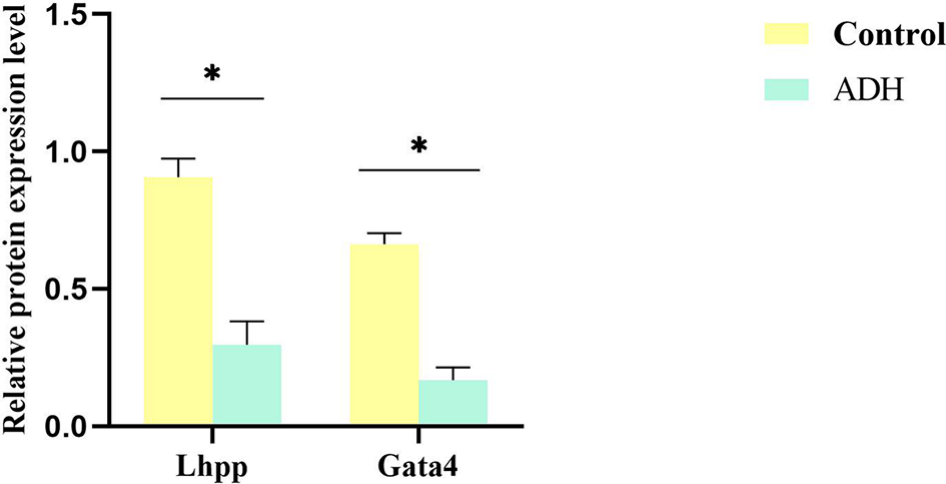
Validation of transcriptome and proteome data from intestinal tissue. A set of DEPs were selected and validated by PRM respectively. Each data point is calculated from averages of biological triplicates. *P < 0.05.

The relative content of GATA4 and LHPP protein in the intestinal tissue samples was detected by PRM method. The results showed that the expression of GATA4 and LHPP in the model mice was down-regulated compared with the control mice. The results of PRM verification were consistent with those of RNA-Seq transcriptome sequencing and TMT analysis (**Figure 9**).

## Discussion

The availability of excellent animal models and the similarity of mutation spectra between hereditary and sporadic diseases contribute to our understanding of disease development and progression (22). In this study, a mouse model of intestinal adenomatous polyp was established by high fat diet combined with AOM/DSS, and the model was evaluated from the aspects of histopathology, transcriptome, proteome and intestinal flora.

Studies have shown that the disease activity index (DAI)of mice fed with high-fat diet is significantly higher than that of mice fed with low-fat diet, and high-fat diet can promote the occurrence of intestinal inflammation in mice (23, 24). In the process of modeling, the body weight of ADH mice decreased significantly, and the body weight of mice decreased in varying degrees after giving DSS in drinking water. The phenomena of depressed mental state, reduced appetite, weight loss, thin stool, prolapse of anus and hematochezia simulated the process of recurrent chronic enteritis and reflected the successful establishment of the ADH model. In this process, the death caused by intestinal obstruction in a very small number of mice may be related to the intolerance of mice to AOM or DSS. The pathology of the colon showed dense glands, sieve pore-like structure, obvious dysplasia of glandular epithelial cells, enlargement of nucleus and pathological mitosis, and chronic inflammatory cell infiltration in the stroma, that was in consistent with the morphology of intestinal tubular adenoma, which accounts for 75% of the incidence of human intestinal adenoma. The above phenomena are in consistent with reports in literatures (25–27).

At present, the success of animal modeling evaluated by histopathology can no longer meet the current demand for the pathogenesis of diseases. In this study, we combined RNA-seq transcriptome sequencing and TMT labeling high-throughput proteome technology to determine the comprehensive and dynamic changes of gene and protein content in intestinal tissue of mice with adenomatous polyp induced by AOM-DSS. A total of 604 genes related to intestinal adenoma were screened by intersection analysis with intestinal adenoma-related targets retrieved from database. These genes are significantly enriched in biological processes such as signal transduction, inflammatory response, negative regulation of apoptotic process and cell proliferation. They are involved in signaling pathways of cancers, PI3K-Akt signaling pathway, MAPK signaling pathway, MicroRNAs in cancer and other signaling pathways. 42 proteins were screened and they are significantly enriched in biological processes such as signal transduction, positive regulation of transcription and apoptotic process. They are involved in signaling pathways such as Regulation of actin cytoskeleton, Leukocyte transendothelial migration and Cell adhesion molecules (CAMs). The results of transcriptome and proteomics analysis showed that 14 genes not only changed significantly at the transcriptional level, but also the proteins encoded by these 14 genes. GO analysis showed that these 14 targets were mainly involved in biological processes such as positive regulation of transcription, DNA-templated, positive regulation of cell migration and signal transduction. The analysis of differential genes and differential protein cross-linking showed that the mouse model of intestinal adenoma induced by AOM/DSS showed similar characteristics to that of human colorectal adenoma at the molecular level.

In addition, the change of intestinal flora has been proved to play an important role in the occurrence and development of many inflammatory intestinal diseases (28–30). Some studies indicated that the change of intestinal flora is a sensitive biological index to judge CRA (31–33). In the ADH mice, the intestinal floral species abundance increased and the microflora diversity decreased, indicating that the model changed the constituents of intestinal flora in mice. In the correlation analysis between differentially expressed proteins and differential bacteria in the model group, four targets (APOC3, GPNMB, GATA4, LHPP) had the highest correlation coefficient with three differential bacteria, namely Candidatus_Saccharimonas, Saccharimonadales, and Bacteroidaceae. It was speculated that they were the core targets and key differential bacteria in the pathogenesis of intestinal adenoma. Bacteroidaceae is a member of the Bacteroides family, which exists widely in the intestinal flora (34). Mice are the most common hosts of Bacteroidaceae (35). Bacteroidaceae may compromise the host defense system, help bacteria to escape immune clearance, and assist in the spread and invasion of bacteria. It is a conditional pathogen (36). In acute necrotizing pancreatitis, the imbalance of intestinal flora leads to the failure of intestinal barrier function, and the content of Candidatus_Saccharimonas bacteria was significantly decreased in the mouse with acute necrotizing pancreatitis (37). HuangY et al. found that the expression levels of cadherin-11, IL-17 α and TLR2 were negatively correlated with the abundance of Candidatus Saccharimonas (38). Other studies have found that the abundance of Candidatus_Saccharimonas is related to the phenotypic utilization efficiency of nitrogen (39). At present, there are few reports about Saccharimonadales. In this study, the abundance of Saccharimonadales in model mice increased significantly, which is speculated to be related to the formation of adenoma, which will be verified by further experiments. The intestinal microflora of normal mice was significantly different from that of the ADH mice. The intestinal probiotics of ADH mice decreased and the pathogenic bacteria increased. The structure and abundance of microflora were similar to those of human intestinal adenoma.

CRA has a high degree of heterogeneity and genomic instability, while the same gene has different regulatory effects on different tumors (40). In order to determine the expression of four targets in CRA, APOC3, GPNMB, GATA4 and LHPP, their expression was analyzed in GEPIA database. The expression of GATA4 and LHPP was consistent with that of the database, and verified by PRM method. The results were highly consistent with the results of RNA-Seq transcriptome sequencing and TMT analysis. At present, there are few reports on LHPP. Previous studies have found that LHPP is related to chronic oxidative stress and mitochondrial dysfunction (41). Studies have shown that the decrease of LHPP increases the level of protein histidine phosphorylation, which leads to uncontrolled growth and diffusion of tumor cells (42, 43). Its downregulation increases the expression and activity of p-AKT and p-PI3K, participates in tumorigenesis, and inhibits the growth and proliferation of colorectal cancer cells by inhibiting the activity of PI3K/AKT signal pathway and promoting the expression of pmur53 (44, 45). GATA4 plays a role in the occurrence and development of tumors. Hellebrekers (46) transferred the expression plasmid of GATA4 into RKO and HCT1116 cells, and found that GATA4 could significantly inhibit the proliferation and migration of colon cancer cells. Agnihotri et al. injected homologous glioma cells with high expression of GATA4 into nude mice and found that the control group with high expression of GATA4 survived with tumor-free, whereas the treatment group died of malignant glioma within 31 ± 7 days after injection (47). They also found that GATA4 could induce the expression of cell cycle inhibitory protein p21 and inhibit the proliferation of cancer cells (48). Therefore, both GATA4 and LHPP play a role similar to tumor suppressor genes. The mutation, deletion or inactivation of these genes can cause malignant transformation of cells and lead to the occurrence of tumors. This finding suggests that GATA4 and LHPP may be the important molecular markers of ADH model and are expected to become the effective serological markers for the diagnosis of intestinal adenomas and potential therapeutic targets. However, the specific mechanism is not clear and needs further study. The data of this study can provide options for further dynamic study of the occurrence and development of CRA, and provide new ideas and basis for the diagnosis, treatment and prevention of CRA. Because the disease itself is the result of the synergistic action of multiple biological factors and their signal transduction pathways, and the bioinformatics data obtained by combinatorial screening is lengthy to a certain extent, in order to obtain the regulatory targets related to the pathogenesis of adenoma, further modeling and analysis is needed.

In conclusion, this study demonstrated that tubular adenoma is an important intermediate link in the process of inflammatory carcinogenesis induced by high fat diet combined with chemical induction. There are similar changes in the intestines of ADH mice and human intestinal adenomas. LHPP and GATA4 could potentially be important molecular pathological markers of intestinal adenoma.

## Data Availability Statement

The datasets presented in this study can be found in online repositories. The names of the repository/repositories and accession number(s) can be found in the article/**Supplementary Material**.

## Ethics Statement

This experiment was approved by the Experimental Animal Ethics Committee of Shanghai University of Traditional Chinese Medicine (Ethics No. YYLAC-2019-042-1).

## Author Contributions

XF contributed to study design and data interpretation. CG and ZC prepared the manuscript. XH and YX contributed to animal rearing. XL and RX contributed to data acquisition and analysis. All authors contributed to the article and approved the submitted version.

## Funding

This work was supported by the National Natural Science Foundation of China (81403360) and the Hospital fund of Yueyang Hospital (2019YYZ08). The funders play no role in data collection and analysis, design, decision to publish, or preparation of the manuscript.

## Conflict of Interest

The authors declare that the research was conducted in the absence of any commercial or financial relationships that could be construed as a potential conflict of interest.

## Publisher’s Note

All claims expressed in this article are solely those of the authors and do not necessarily represent those of their affiliated organizations, or those of the publisher, the editors and the reviewers. Any product that may be evaluated in this article, or claim that may be made by its manufacturer, is not guaranteed or endorsed by the publisher.
